# Molecular Characterization of *Staphylococcus aureus* Isolated from Raw Milk and Humans in Eastern Tanzania: Genetic Diversity and Inter-Host Transmission

**DOI:** 10.3390/microorganisms11061505

**Published:** 2023-06-05

**Authors:** Tutu Mzee, Happiness Kumburu, Theckla Kazimoto, Pimlapas Leekitcharoenphon, Marco van Zwetselaar, Rose Masalu, Tarsis Mlaganile, Tolbert Sonda, Boaz Wadugu, Ignass Mushi, Frank M. Aarestrup, Mecky Matee

**Affiliations:** 1 Ifakara Health Institute, Bagamoyo Branch, Bagamoyo P.O. Box 74, Tanzania; 2 Department of Molecular Biology and Biotechnology, University of Dar es Salaam, Dar es Salaam P.O. Box 35179, Tanzania; 3 Kilimanjaro Clinical Research Institute, Moshi P.O. Box 2236, Tanzania; 4 Research Group for Genomic Epidemiology, National Food Institute, Technical University of Denmark, Kemitorvet, DK 2800 Kgs. Lyngby, Denmark; 5 Department of Microbiology and Immunology, School of Medicine, Muhimbili University of Health and Allied Sciences, Dar es Salaam P.O. Box 65001, Tanzania

**Keywords:** *Staphylococcus aureus*, antibiotic resistance, genotyping, whole genome sequencing, virulence factors, asymptomatic mastitis, Tanzania

## Abstract

*Staphylococcus aureus* is a common cause of infection in humans and animals, including bovine mastitis, globally. The objective of this study was to genetically characterize a collection of *S. aureus* isolates recovered from milk and nasal swabs from humans with and without animal contact (bovine = 43, human = 12). Using whole genome sequencing (NextSeq550), isolates were sequence typed, screened for antimicrobial resistance and virulence genes and examined for possible inter-species host transmission. Multi locus sequence typing (MLST) and single nucleotide polymorphism (SNP)-based phylogeny revealed 14 different sequence types, including the following six novel sequence types: ST7840, 7841, 7845, 7846, 7847, and 7848. The SNP tree confirmed that MLST clustering occurred most commonly within CC97, CC5477, and CC152. ResFinder analysis revealed five common antibiotic resistance genes, namely *tet*(K), *blaZ*, *dfrG*, *erm*©, and *str*, encoding for different antibiotics. *mecA* was discovered in one human isolate only. Multidrug resistance was observed in 25% of the isolates, predominantly in CC152 (7/8) and CC121 (3/4). Known bovine *S. aureus* (CC97) were collected in humans and known human *S. aureus* lineages (CC152) were collected in cattle; additionally, when these were compared to bovine-isolated CC97 and human-isolated CC152, respectively, no genetic distinction could be observed. This is suggestive of inter-host transmission and supports the need for surveillance of the human–animal interface.

## 1. Introduction

*Staphylococcus aureus* is a commensal bacterium found on human and animal skin as well as mucous membranes. As a pathogen, *S. aureus* is associated with causing a wide range of infections ranging from mild skin infections to more life-threatening infections such as pneumonia, endocarditis, bloodstream infections, and food poisoning [1,2]. *S. aureus* is also the leading cause of mastitis, one of the most important and economically costly infections in dairy cattle [3]. The success of *S. aureus* as a pathogen might have emerged as a result of massive control programs against *Streptococcus agalactiae*, a bacterium associated with subclinical mastitis, resulting in low milk quality and low yield [4]. The elimination of *Streptococcus agalactiae* in dairy production farms has notably facilitated the rise of *S. aureus*, which similarly causes subclinical infections associated with permanent production losses and is very difficult to cure [3].

It was previously assumed that there were a number of strains responsible for most global mastitis infections [5,6], and CC97 has been suggested as the most common mastitis-causing bovine-adapted *S. aureus* strain [5,7], with its ability to avoid the bovine immune response attributable to its dominance [7,8]. Nevertheless, evidence of herd-specific *S. aureus* strains causing mastitis has also been reported in different studies [7,9], indicating differences in clonal complexes, virulence factors, and antimicrobial conferring genes in *S. aureus* collected from different sources and geographic areas [7]. In African studies this was also apparent, however most of the strains reported were novel, belonging to CC97 [10,11]; more recent studies have reported dominance of ST5477 and 152 in bovine-originating strains isolated in eastern Africa [12]. ST152 was first isolated in Europe but was later described as a dominant human isolate in Africa [13,14]. The dominance of a human clone (ST152) among bovine-isolated strains is indicative of animal–human transmission, which calls for further examination [15].

*S. aureus* has the ability to produce exotoxins that have haemolytic and cytotoxic activities, which hinder phagocytosis, facilitating infection in the host. The most notable exotoxins in this bacterium belong to the Staphylococcal enterotoxins (SEs) family, which are heat stable potent toxins, also responsible for non-specific T-cell proliferation. These virulence factors are associated with skin infection (including mastitis), food poisoning, allergic and autoimmune diseases [16,17]. Panton–Valentine leukocidin (PVL) is another virulence factor produced by the bacterium, capable of tissue and cell necrosis, especially the destruction of leukocytes. SEs and PVL are the most potent virulence factors, playing a pivotal role in the initiation and pathogenesis of the disease [18,19]. *Staphylococcus* protein A is also an important virulence determinant; it is a surface Ig-binding protein whose function is to capture IgG molecules, preventing phagocytosis of bacterial cells from happening, which results in the invasion of the host’s immune response [20]. The *Spa* gene encoding for the surface protein A is also frequently used for genotyping purposes, and typing is usually based on the sequence variation as well as the number of tandem repeats in the X region of the gene [20,21].

Varying antimicrobial resistance among *S. aureus* isolates originating from humans and animals has been reported [22]. However, generally, bovine-originating *S. aureus* exhibits fewer resistance genes, mostly associated with resistance against penicillin caused by the *blaZ* gene [5,11]. Notably, large resistance variations between studies and between countries have been reported over time. Recent bovine-associated *S. aureus* strains in Africa carry a wider range of resistance genes [12,23]. Multidrug resistance has been increasing globally, which is considered a public health concern [24]. Several recent investigations reported the emergence of multidrug-resistant bacterial pathogens from different origins, which increase the necessity of antibiotics stewardship and other surveillance methods, as well as the routine application of antimicrobial susceptibility testing to detect antibiotic resistance as well as MDR strains [11,25,26].

The lack of information about *S. aureus* characterization, associated antimicrobial genes and virulence factors in the country highlights the need to examine the bacterium in different reservoirs. Taking the One Health approach, this study assessed isolates from humans and cattle milk in different parts of Tanzania. The objectives were to (i) investigate the distribution of sequence types circulating in eastern Tanzania using MLST and *Spa* typing, (ii) screen for antimicrobial resistance and virulence genes, and (iii) evaluate inter-host transmission and genetic relatedness of *S. aureus* isolates recovered from humans and animals in the study area.

## 2. Materials and Methods

### 2.1. Ethical Approval

The Vice-Chancellor of the University of Dar es Salaam issued a research permit letter on behalf of the National Institute of Medical Research (NIMR) and the Tanzania Commission for Science and Technology (COSTECH) with reference no. AB3/12 (B). Further permission to carry out this study was granted by the Executive Directors of the Handeni, Bagamoyo, and Morogoro urban districts. In addition, written consent was provided by all human participants prior to being sampled.

### 2.2. Study Site

Three eastern Tanzanian districts were included in this study. Bagamoyo (Pwani region) and Morogoro urban (Morogoro region) were categorised as semi-urban districts, and Handeni (Tanga region) was categorised as a rural district.

### 2.3. Study Design

This was a cross-sectional study conducted between September 2017 and December 2017 in the Eastern zone of Tanzania. The study involved the Tanga, Coast, Morogoro, and Dar es Salaam regions. Tanga, Coast, and Morogoro regions are categorized as moderately dense livestock-keeping communities (Ministry of Livestock and Fisheries report 2015/2016 [27]). Eighty-four *S. aureus* isolates were collected from the raw milk of seemingly healthy cattle from 16 dairy farms in the study area (out of 43 included in the study). Six human *S. aureus* nasal swab isolates from humans with animal contact who worked or resided on the sampled dairy farms were taken and all were included in the study. Further samples from humans with no animal contact were taken in Dar es Salaam, the commercial capital of Tanzania. Out of 20 *S. aureus* isolates identified from this group, 6 were included in this study.

### 2.4. Sample Collection

Dairy farms housing between 20 and 500 cattle were included. The sample size for each region was calculated by using a stratified method, whereby the three regions of interest, i.e., Morogoro, Coast, and Tanga, acted as strata. The estimated population of dairy cattle was established, and an assumption was made of constant prevalence of *S. aureus* of 50% across all regions with a precision within 10% of the true prevalence. A 95% confidence interval was used.

At district level, extension officers provided lists of farms in the given area. Purposive sampling was used to choose qualifying farms to be interviewed and sampled. The study team visited the chosen farms to ask for consent and to determine the exact number of animals on each farm. This information was used to calculate the exact sample size to be collected on each farm. This was calculated considering the number of animals on the farm, the number of animals on all farms to be sampled, and the calculated sample size in the particular region.

About 8–10 mL of midstream milk was collected in sterile Falcon tubes from individual cattle during the milking process. Nasal swabs from people residing or working on the same farm were collected consecutively. After obtaining their consent for participation, sterile cotton swabs were dipped in distilled water and gently rubbed against both inner nares of the participant’s nose. Samples for the group of people with no animal contact were collected from drug addicts residing in the commercial city of Dar es Salaam. After obtaining consent and completing a questionnaire to ensure the participant had had no contact with any animal in the past 12 months, sampling took place using the same procedures as in people with animal contact. The samples were then dipped into Stuart Transport Medium in 15 mL Falcon tubes. All types of samples were kept in a cooler box at temperatures ranging from 4–8 °C before being transported to the laboratory for further analysis.

### 2.5. Identification

Milk samples were pre-enriched with buffered peptone water at a 9:1 ratio (9 mL of peptone water to 1 mL of milk sample), and the mixture was incubated for 24 h at 37 °C [28]. After the milk enrichment step, all samples were treated the same, and were cultured aerobically at 35 °C for 18–24 h on Columbia 5% Sheep Blood Agar (Biorad, Marnes-la-Coquette, France) and Mannitol Salt Agar (Oxoid Ltd., Basingstoke, UK). *S. aureus* colonies were presumptively identified by morphology and haemolysis on Blood Agar, Mannitol fermentation (yellowish colonies), Gram staining and catalase production. Identification of *S. aureus* was confirmed by a tube coagulase test (BD BBL Coagulase plasma, Rabbit) [25,29].

### 2.6. DNA Extraction and Whole Genome Sequencing

Genomic DNA was extracted using a QIAamp DNA min kit (Qiagen GmbH, Hilden, Germany). The quality and quantity of genomic DNA were confirmed using a Qubit 2.0 fluorometer, (Thermal Fisher Scientific, Waltham, MA, USA). Library preparation (dual indexing) was performed using an Illumina DNA prep kit, (Illumina Inc., San Diego, CA, USA). Whole genome sequencing of the library was completed on an Illumina NextSeq 500 platform using a paired-end 2 × 150 bp protocol.

### 2.7. Bioinformatic Analysis

Species identification, multilocus sequence typing (MLST), *Spa* typing, and identification of antimicrobial resistance and virulence genes were carried out using Center for Genomic Epidemiology web-based tools (Bortolaia et al., 2020, Larsen et al., 2012, Carattoli et al., 2014). All tools are available online at http://cge.cbs.dtu.dk/services (accessed on 22 April 2023). Raw sequence data have been submitted to the European Nucleotide Archive (http://www.ebi.ac.uk/ena (accessed on 17 February 2023)) under study accession No. PRJEB59926.

## 3. Results

A total of 43 *S. aureus* were isolated from cows’ milk samples from the main local milk-supplying farms in different Tanzanian regions. Twelve of the *S. aureus* isolates included were of human origin, six of which originated from people residing at or working on the same dairy farms as the sampled cattle and six from people with no animal contact (drug addicts) residing in Dar es Salaam. The isolates were sequenced using NextSeq 550 in order to determine sequence types, screen for antimicrobial resistance and virulence genes. Further genetic relatedness between the animal- and human-isolated *S. aureus* and evidence of possible inter-host transfer was evaluated. A summary of the findings can be found in Table 1 and Table 2.

### 3.1. Isolates Characteristics, Species Identification, and Multilocus Sequence Typing

Of the 43 milk-originating isolates, nine were from the Tanga region, 21 from Bagamoyo, and 13 from the Morogoro region. Multilocus sequence typing revealed 14 different sequence types among the 55 isolates. The most frequent STs were ST7846 (21.8%), 97 (16.36%), 152 (14.54%), and 5477 (12.72%), 121 (7.3%), 7841 (7.3%) and 7840 (5.45%). Other sequence types occurred twice or singly. A high diversity of STs was observed in all regions, especially Bagamoyo. The study detected five novel sequence types: ST7840, 7841, 7845, 7846, 7847, and 7848. Morogoro was dominated by two new STs, 7840 and 7846, as well as ST8 and 152, which have previously been reported elsewhere. Sequence type 5477 was only observed in milk samples from Tanga and Bagamoyo (Table 1a,b).

### 3.2. Antimicrobial Resistance, Virulence, Leukocidin Genes, and Spa Typing

ResFinder analysis revealed a limited number of antibiotic resistance genes among the *S. aureus* in this study, including *tet*(K), *blaZ*, *dfrG*, *erm*(C), *str*, *qacG* and *fosB* encoding for tetracycline, penicillin, trimethoprim, macrolide, lincosamide, streptogramin B (MLSB), and multidrug efflux pumps. *mecA* was discovered in one of the 55 isolates included in the study. Specificity of resistance genes, virulence factors, toxin genes, and leukocidin genes in different sequence types could also be observed (Table 2). ST7846 was isolated from cattle and humans (BH00403) in the Morogoro and Pwani regions. The isolates uniformly conferred the penicillin resistance *blaZ* gene, whereas the majority also conferred the *str* and *tet*(K) genes, exhibiting either the *blaZ/tetK* or *blaZ/str* combination. None of the isolates exhibited all three genes together. The sequence type also homogeneously possessed the *lukD/E* leukocidin genes, as well as the *aur*, *hlgA*, *hlgB*, *hlgC*, and *splA/B* virulence genes. All isolates in this sequence type belonged to *Spa* type t1236.

ST97 isolates were also collected in humans (DADST084, DADST088) and milk in Tanga, Pwani, and Dar es Salaam. All isolates belonging to this sequence type possessed the *blaZ* resistance gene, further the *blaZ/tetK* combination was also frequently observed. Isolates in this group exhibited *lukD/E* leukocidin genes as well as *aur*, *hlgA*, *hlgB*, *hlgC*, and *splA/B* virulence genes which were *Spa* typed t9432. Four within the ST97 sequence type possessed the *splE* virulence gene, which were also exclusively screened with the sak and scn toxic genes (*Spa* typed t267).

The ST5477 isolates in this study were of bovine origin, collected in the Tanga and Pwani regions, all of which encoded for the *blaZ* gene. Moreover, all ST5477 possessed *aur*, *edinB*, *hlgA/C*, and *splA/B* virulence genes, and a few exhibited an additional *hlgB* gene. This ST was further prone to the egc-cluster enterotoxin genes (*seg*, *sei*, *sem*, *seo*, and *sen*) and homogeneously encoded for the toxin shock *tst* gene. Further, all ST5477 encoded for only the *lukE* leukocidin gene, with the exception of one that exhibited the *lukD/E* combination. Only two isolates in this group were assigned *Spa* types, namely t18852 and t18853.

The novel ST7841 (*Spa* type t042) was exclusive to bovine isolates collected in the Pwani region. This sequence type exhibited *blaZ* and *str* resistance as well as *aur, hlgA*, *hlgB*, *hlgC*, *splA*, *splB*, and *splE* virulence genes. All were screened with *lukD/E* leukocidin genes and not toxin genes.

Novel ST7840 (*Spa* type t1398) isolates were also of bovine origin and exclusively isolated in the Morogoro region. All three exhibited the *lukD* leukocidin gene as well as *aur*, *edinB*, *hlgA*, *hlgB*, *hlgC*, *splA*, and *splB* virulence genes coupled with a range of egc-cluster enterotoxin genes. Two of the three ST7841 isolates were not detected with any resistance genes, and the one that was detected with one was screened with the *erm*(C) resistance gene. Furthermore, the novel ST7845 was another bovine-exclusive ST collected in the Pwani region. Two isolates belonged to this ST, and neither presented any toxin genes. One of the two was screened with the *blaZ* gene, whereas the other was detected with *blaZ*/*str*/*tet*(*K*) resistance genes. Both presented *aur*, *edinB*, *hlgA, hlgB*, and *hlgC* virulence genes and *lukE* as their leukocidin gene.

ST152 was observed in both human- and cattle-originating samples collected in the Tanga, Pwani, and Morogoro regions. Regardless of origin, the most common resistance genes in this sequence type included *blaZ*, *dfrG*, *erm*(*C*), and *tet*(*K*). The majority of the isolates in this ST encoded for *edinB*, *hlgA*, and *hlgB* virulence genes and exclusively for the *sak* and *scn* toxic genes. Additionally, all ST152 were PVL-positive screened with the *lukF/S-PV* genes. All of the ST152 were *Spa* typed t355, with exception of one that was typed t11429.

Interestingly, although three of the four ST121 isolates were of human origin collected in Dar es Salaam, and one of bovine origin (TC00402) that was collected in Tanga, all exhibited similar characteristics. The majority encoded for *blaZ*, *dfrG*, and *erm*(*C*) resistance genes. The sequence type was homogeneously *Spa* typed t272, whereby most encoded for *aur*, *edinC*, *hlgA*, *hlgB*, *hlgC*, and *splA* virulence genes, and all four encoded for a combination of *sak/scn* and egc-cluster (*seg*, *sei*, *sem*, *seo*, and *sen*) genes as well as *eta/b* exfoliating genes. Moreover, none of the ST121 encoded for any of the leukocidin genes (Table 2).

### 3.3. Single Nucleotide Polymorphisms (SNPs)

**Figure 1:**
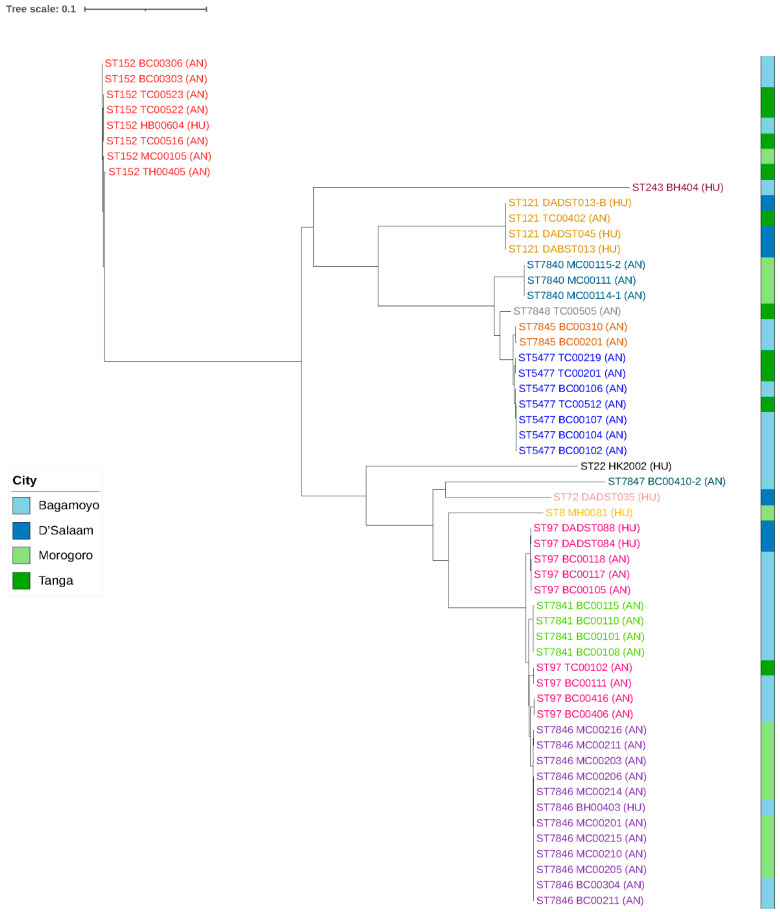
SNP tree of 55 *S. aureus* genomes constructed using CSI phylogeny. AN: Isolates collected from cows, HU: Isolates collected from humans. Clustering STs are colour-coded. The colour-coded bar represents the regions in which the isolates were collected from.

The mapping of the raw reads of these 55 genomes to the reference genome, *S. aureus* 55-99-44 (NCBI ID CP024998.1), detected 20,614 qualified SNPs which were used to construct a maximum likelihood SNP tree (Figure 1). The SPN tree is composed of several different clusters of closely related isolates. The ST152 strains composed a stand-alone cluster of eight isolates originating from both humans and animals. Two larger branches emerging from a common ancestral point were also observed, each consisting of several smaller clusters with different sequence types. STs 7840, 7845, 7848, and 5477, which belong to the same cluster complex (CC5477), formed a cluster emerging from a common point. The cluster comprised exclusively of closely related bovine strains. These CC5477 strains further clustered with a ST121 branch that consisted of three human and one bovine isolate collected in Dar es Salaam and Tanga, respectively (Figure 1).

The subsequent cluster comprised strains belonging to CC97, composed of ST97, ST7841 and ST7846. The majority of the isolates in the CC97 cluster were of bovine origin, but a number of human-originating isolates were also represented. ST97 was represented twice in the same cluster. In the first instance, five isolates of bovine and human origin clustered together in a sub-cluster. In the second instance, four bovine isolates clustered together in a sub-cluster. The largest sub-cluster was ST7846, that is composed of 12 bovine- and human-originating isolates; sub-cluster ST7841 comprised of four bovine strains, all collected in Bagamoyo (Pwani). Generally, the SNP difference among clustered strains was between 2 and 10 (Supplementary Material (SNP matrices)).

## 4. Discussion

To establish targeted interventions in human and animal health and reduce the effects of *S. aureus*, it is important to understand its occurrence, evolution and transmission within and between humans and animals. The present study was conducted to provide information on sequence types, virulence and antimicrobial associated genes found in *S. aureus* isolated from cows’ milk and humans working on the same dairy farms, as well as in humans with no animal contact who were sampled in eastern Tanzania.

We observed a high diversity of sequence types; however, we could categorize them into three main clusters. The largest cluster was ST97, which clustered with novel STs belonging to the clonal complex CC97. Isolates of this cluster were found in humans with no animal contact sampled in Dar es Salaam as well as in milk sampled from Tanga, Bagamoyo (Pwani) and Morogoro. The fact that humans with no animal contact were colonized by a known bovine ST strain (ST97) may be explained by a secondary manifestation from a human with animal contact. The human isolates in question are clustered with milk samples from Bagamoyo (BC00118, BC00117, and BC00105), which is located about 60 km from where the human samples were collected. Human contact between the two towns is very high, further supporting the theory of secondary manifestation from a human with animal contact. CC97 has long been established as a bovine-specific lineage; however, there have also been reports of the clonal complex being found in humans [30,31].

ST5477 clustered with novel STs ST7840, ST7845, and ST7848, and the cluster was exclusively populated by bovine originating *S. aureus*, indicating that it might be a bovine-specific clonal complex. The isolates in the cluster were collected in all three regions (i.e., Pwani, Morogoro, and Tanga), which is suggestive of the wide spread of the clonal complex in Tanzania.

ST5477 has previously been reported once in bovine-associated isolates in Rwanda [12]. The recent emergence of this sequence type in two neighboring countries cannot be explained; however, the most probable reason may be attributed to cattle trade between Rwanda and Tanzania. We have managed to build on the information gathered by the Rwandese study, and more sequence types closely related to ST5477 were gathered. This observation calls for further investigation into the ecology, evolution, and epidemiology of this and other clonal complexes, in order to establish the most dominant African bovine *S. aureus* lineages.

ST152 was a standalone branch that did not cluster with the rest of the isolates collected in the study. The eight isolates represented in this cluster stemmed from humans and dairy cattle and were collected from all three participating regions. Unfortunately, the study did not manage to obtain isolates from humans and cattle residing on the same farm; nevertheless, no genetic distinction was observed within the cluster, which is suggestive of host transfer between the two reservoirs. The majority of the ST152 were categorized as *Spa* type t355 and PVL+ MSSA, which is consistent with the majority of reports from the African continent [14,32].

**Figure 2:**
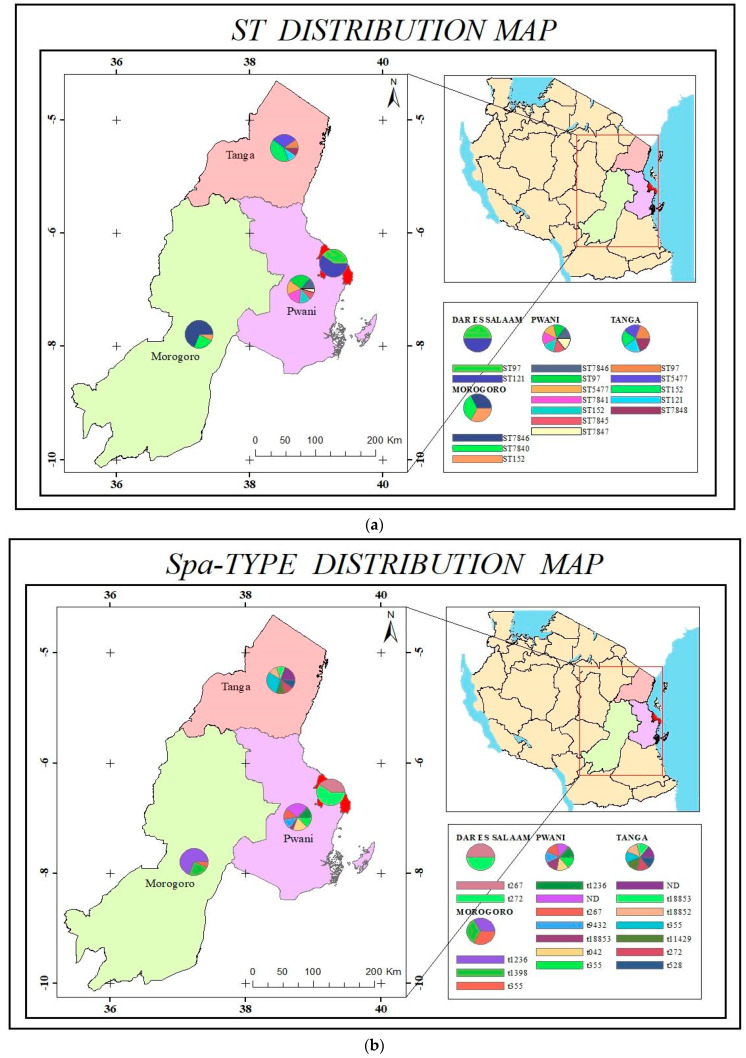
(**a**) ST diversity and distribution within the study area; (**b**) *Spa* type diversity and distribution within the study area.

In general, the data suggest the existence of contagious transmission within, as well as between, regions. For instance, novel STs 7841 and 78,445 were found only in Bagamoyo and Morogoro, respectively, while other STs were observed in more than one region. The highest diversity among the isolates was observed in Bagamoyo (Pwani), although the region contributed the majority of the isolates; high diversity in the area (Figure 2a,b) may also be attributed to the region’s near proximity to the commercial capital city, as well as being the gateway to all northern regions. To cater for the high demand for milk and red meat in Dar es Salaam, a large influx of cattle from different parts of the country may pass through Bagamoyo to get to Dar es Salaam, creating a reservoir of different *S. aureus* sequence types in the area.

Five resistant conferring genes were predominantly observed in the dataset. *blaZ*, the gene conferring for penicillin resistance, was prevalent, at 84%, which is a common occurrence among bovine *S. aureus* as the antibiotic is frequently used to treat intramammary infections in cattle [12,33]. The streptomycin-resistant gene *str* was only detected in bovine *S. aureus* and *tetK*, in which it was prevalent at 23% and 24%, respectively. This can be explained by the high use of Penistrep and tetracycline to treat various animal infectionsin the country [34]. Multidrug resistance, which was defined as harboring three or more resistance genes, was identified in 25% of the collected samples, most of which were observed in ST152 and ST121. Both ST152 and ST121 were populated by human- and bovine-originating isolates and exhibited *ermC* and *drfG* genes conferring for erythromycin and trimethoprim resistance. The two STs have previously been isolated from both human and animal sources, supporting the hypothesis that the sequence types might have moved from human to animals, and hence presenting resistance to antibiotics more frequently used in humans [12,35]. Furthermore, none of the bovine-originating isolates were detected with *mecA* or *mecC*, hence all samples were categorized as MSSA. The only MRSA was detected in a dairy farm worker, which was categorized as ST8, a rather successful *S. aureus* lineage from which a number of MRSA have emerged. Additionally, the ST has also been observed in animals such as horses and cattle, posing potential threat of cross infection between humans and animals [31,36]. Virulence genes play a pivotal role in determining and developing infection, as well as the subsequent spread of infection within the same host species or between different hosts. Clear sequence type specificity in virulence factors could be observed. Hemolysin encoding genes were very frequently detected across regions, sequence types, and host species (*hlgA* 100%, *hlgB* 83.6%, and *hlgC* 83.6%). Leukotoxin encoding genes were also detected in the majority of the isolates, mostly exhibiting the *lukD/E* combination (50.9%), which is associated with the ability to target lymphocytes of a broad host range, markedly facilitating *S. aureus* pathogenicity [37]. Moreover, the *lukED/hlgAB* combination observed in 49% of the isolates is reported to be highly functional in erythrocyte binding and hemolysis, further facilitating the strain’s pathogenic capacities.

ST97, ST7846, ST7841, ST152, ST7845, and ST7847 were not detected with enterotoxins. Nevertheless, none of the sequence types that did exhibit enterotoxins (65%) were detected with classic sea–see genes, all exhibited seg to seo enterotoxins, regardless of human or bovine origin. It is well known that 95% of food poisoning is caused by enterotoxins, which have the ability to retain their biological and immunological activities following pasteurization, as well as exposure to gastrointestinal protease [38]. Toxin shock encoding gene was detected in 16% in both human and bovine isolates; however, in bovine-originating samples, the gene was exclusively observed in ST5477 (12.7%). High detection levels of enterotoxins in the isolates increases the possibility of food poisoning through consumption of milk or milk products. Enterotoxins are further considered as a potential environmental pathogenic contaminant, even after leaving the human body [16,38]. In examining the genetic relatedness and potential host transfer, nine of the 12 human samples clustered with bovine isolates. Isolates from both hosts, when in a cluster, were genetically indistinguishable, exhibiting the same STs and *Spa* types. The predominance of *lukD/E* virulence factors in the isolates is also indicative of them being of bovine origin. Although *lukD/E* is not limited to bovine-associated lineages, they are known to have selective advantage in the bovine host [11]. Further, when bovine-originating isolates clustered with human-associated lineages such as CC152 and CC121, they were also genetically indistinguishable, further exhibiting immune evasion clusters (scn, sak) that are uncommonly found in animals and are primarily needed for immune evasion in humans [39,40]. These observations strongly suggest *S. aureus* host transfer from bovine to human and vice versa. Some evidence proposes that some *S. aureus* are only capable of colonizing and infecting certain host species; nonetheless other lineages are non-specific. It has further been proven that *S. aureus* is highly adaptive to new environments through gene transfer and mutation [30]. It is therefore important that the interface between animals and humans is under constant surveillance in order to detect any population change in a timely manner.

## 5. Conclusions

The study presents an insight into antimicrobial conferring genes, virulence genes, and genetic diversity of *S. aureus* collected from milk and human samples in different areas around eastern Tanzania. We observed high diversity of *S. aureus* among human and bovine-originating isolates. Fourteen ST were documented, of which six were novel STs. Most isolates could, however, be grouped into three dominant clonal complexes, namely CC152, CC5477 and CC97. The low occurrence of antibiotic resistance conferring genes and the lack of PTSA genes in the majority of the bovine-originating isolates suggests that human health risk caused by bovine *S. aureus* is low. Nevertheless, the findings did suggest inter-host transmission, as known human lineages were collected in bovine samples and known bovine lineages were collected in humans. These isolates were genotypicaly indistinguishable when clustered with human- and bovine-specific clonal complexes. These finding therefore suggest that the two hosts can act as each other’s reservoirs for antibiotic resistance and virulence genes, adopting pathogenic factors which, in turn, supports the need for rigorous surveillance of the bovine–human interface to track *S. aureus* population change in a timely manner.

## Figures and Tables

**Table 1:** (a) Multilocus sequence typing of *S. aureus* genomes collected from cows’ milk from local farms in four regions of Tanzania; (b) Multilocus sequence typing of *S. aureus* genomes collected from nasal swabs of people with and without animal contact.

(a)				
Sample Type	Source	Region	No. of Isolates	Sequence Type (n)
Milk	Cow	Tanga	9	ST97 (1)
				ST5477 (3)
				ST121 (1)
				ST7848 (1)
				ST152 (3)
Milk	Cow	Bagamoyo	21	ST7841 (4)
				ST5477 (5)
				ST97 (5)
				ST7845 (2)
				ST7846 (2)
				ST152 (2)
				ST7847 (1)
Milk	Cow	Morogoro	13	ST152 (1)
				ST7840 (3)
				ST7846 (9)
**(b)**				
**Sample Type**	**Source**	**Region**	**No. of Isolates**	**Sequence Type (n)**
Nasal Swab	Human	Bagamoyo	4	ST22 (1)
				ST7846 (1)
				ST243 (1)
				ST152 (1)
		Tanga	1	ST152
		Morogoro	1	ST8
		Dar es Salaam	6	ST72 (1)
				ST121 (3)
				ST97 (2)

**Table 2:** Antimicrobial resistance genes, virulence genes, leukocide genes, and *Spa* typing of *S. aureus* collected from human nasal swabs and cows’ milk from four regions in Tanzania.

Sample ID	Origin	Region	ST	CC	AMR Genes	Virulence Genes	Toxin Genes	Leukocide Genes	*Spa* Type
BC00211	M	BAG			*blaZ*, *str*	*aur*, *hlgA*, *hlgB*, *hlgC*, *splA*, *splB*	*ND*	*lukD*, *lukE*	*t1236*
BC00304	M	BAG			*blaZ*, *str*	*aur*, *hlgA*, *hlgB*, *hlgC*, *splA*, *splB*	*ND*	*lukD*, *lukE*	*t1236*
MC00201	M	MOR			*blaZ*, *tet*(*K*)	*aur*, *hlgA*, *hlgB*, *hlgC*, *splA*, *splB*	*ND*	*lukD*, *lukE*	*t1236*
MC00203	M	MOR			*blaZ*	*aur*, *hlgA*, *hlgB*, *hlgC*, *splA*, *splB*	*ND*	*lukD*, *lukE*	*t1236*
MC00205	M	MOR			*blaZ*, *str*	*aur*, *hlgA*, *hlgB*, *hlgC*, *splA*, *splB*	*ND*	*lukD*, *lukE*	*t1236*
MC00210	M	MOR			*blaZ*, *str*	*aur*, *hlgA*, *hlgB*, *hlgC*, *splA*, *splB*	*ND*	*lukD*, *lukE*	*t1236*
MC00211	M	MOR	7846	97	*blaZ*	*aur*, *hlgA*, *hlgB*, *hlgC*, *splA*, *splB*	*ND*	*lukD*, *lukE*	*t1236*
MC00214	M	MOR			*blaZ*, *str*	*aur*, *hlgA*, *hlgB*, *hlgC*, *splA*, *splB*	*ND*	*lukD*, *lukE*	*t1236*
MC00215	M	MOR			*blaZ*, *str*	*aur*, *hlgA*, *hlgB*, *hlgC*, *splA*, *splB*	*ND*	*lukD*, *lukE*	*t1236*
MC00216	M	MOR			*blaZ*	*aur*, *hlgA*, *hlgB*, *hlgC*, *splA*, *splB*	*ND*	*lukD*, *lukE*	*t1236*
MC00206	M	MOR			*blaZ*, *str*	*aur*, *hlgA*, *hlgB*, *hlgC*, *splA*, *splB*	*ND*	*lukD*, *lukE*	*t1236*
BH00403	HWA	BAG			*blaZ*, *tet*(*K*)	*aur*, *hlgA*, *hlgB*, *hlgC*, *splA*, *splB*	*ND*	*lukD*, *lukE*	*t1236*
TC00102	M	TAN			*blaZ*	*aur*, *hlgA*, *hlgB*, *hlgC*, *splA*, *splB*	*ND*	*lukD*, *lukE*	*ND*
BC00105	M	BAG			*blaZ*, *tet*(*K*)	*aur*, *hlgA*, *hlgB*, *hlgC*, *splA*, *splB*, *splE*	*sak*, *scn*	*lukD*, *lukE*	*t267*
BC00117	M	BAG			*blaZ*, *tet*(*K*)	*aur*, *hlgA*, *hlgB*, *hlgC*, *splA*, *splB*, *splE*	*sak*, *scn*	*lukD*, *lukE*	*t267*
BC00118	M	BAG			*blaZ*, *tet*(*K*)	*aur*, *hlgA*, *hlgB*, *hlgC*, *splA*, *splB*, *splE*	*sak*, *scn*	*lukD*, *lukE*	*t267*
BC00406	M	BAG	97	97	*blaZ*, *qacG*, *tet*(*K*)	*aur*, *hlgA*, *hlgB*, *hlgC*, *splA*, *splB*	*ND*	*lukD*, *lukE*	*t9432*
BC00416	M	BAG			*blaZ*, *qacG*, *tet*(*K*)	*aur*, *hlgA*, *hlgB*, *hlgC*, *splA*, *splB*	*ND*	*lukD*, *lukE*	*t9432*
BC00111	M	BAG			*blaZ*	*aur*, *hlgA*, *hlgB*, *hlgC*, *splA*, *splB*	*ND*	*lukD*, *lukE*	*t9432*
DADST084	HNA	DSM			*blaZ*	*aur*, *hlgA*, *hlgB*, *hlgC*, *splA*, *splB*, *splE*	*sak*, *scn*	*lukD*, *lukE*	*t267*
DADST088	HNA	DSM			*blaZ*	*aur*, *hlgA*, *hlgB*, *hlgC*, *splA*, *splB*, *splE*	*sak*, *scn*	*lukD*, *lukE*	*t267*
TC00201	M	TAN			*blaZ*, *str*	*aur*, *edinB*, *hlgA*, *hlgB*, *hlgC*, *splA*, *splB*	*sei*, *sem*, *sen*, *tst*	*lukE*	*t18853*
TC00219	M	TAN			*blaZ*, *str*	*aur*, *edinB*, *hlgA*, *hlgB*, *hlgC*, *splA*, *splB*	*sei*, *sem*, *sen*, *seo*, *tst*	*lukE*	*t18852*
TC00512	M	TAN			*blaZ*	*aur*, *edinB*, *hlgA*, *hlgC*, *splA*, *splB*	*sei*, *sem*, *sen*, *seu*, *tst*	*lukD*, *lukE*	*ND*
BC00102	M	BAG	5477	5477	*blaZ*	*aur*, *edinB*, *hlgA*, *hlgC*, *splA*, *splB*	*sei*, *sem*, *sen*, *seo*, *seu*, *tst*	*lukE*	*ND*
BC00104	M	BAG			*blaZ*	*aur*, *edinB*, *hlgA*, *hlgC*, *splA*, *splB*	*sei*, *sem*, *seu*, *tst*	*lukE*	*ND*
BC00106	M	BAG			*blaZ*	*aur*, *edinB*, *hlgA*, *hlgC*, *splA*, *splB*	*sei*, *sem*, *seo*, *tst*	*lukE*	*ND*
BC00107	M	BAG			*blaZ*	*aur*, *edinB*, *hlgA*, *hlgC*, *splA*, *splB*	*sei*, *sem*, *sen*, *seo*, *tst*	*lukE*	*ND*
BC00108	M	BAG			*blaZ*, *str*	*aur*, *hlgA*, *hlgB*, *hlgC*, *splA*, *splB*, *splE*	*ND*	*lukD*, *lukE*	*t042*
BC00110	M	BAG			*blaZ*, *str*	*aur*, *hlgA*, *hlgB*, *hlgC*, *splA*, *splB*, *splE*	*ND*	*lukD*, *lukE*	*t042*
BC00101	M	BAG	7841	97	*str*	*aur*, *hlgA*, *hlgB*, *hlgC*, *splA*, *splB*, *splE*	*ND*	*lukD*, *lukE*	*t042*
BC00115	M	BAG			*blaZ*	*aur*, *hlgA*, *hlgB*, *hlgC*, *splA*, *splB*	*ND*	*lukD*, *lukE*	*t042*
MC00111	M	MOR			*erm*(*C*)	*aur*, *edinB*, *hlgA*, *hlgC*, *splA*, *splB*	*sei*, *sem*, *seu*	*lukE*	*t1398*
MC00114	M	MOR	7840	5477-like	*ND*	*aur*, *edinB*, *hlgA*, *hlgB*, *hlgC*, *splA*, *splB*	*sei*	*lukE*	*t1398*
MC00115	M	MOR			*ND*	*aur*, *edinB*, *hlgA*, *hlgC*, *splA*, *splB*	*sei*, *sem*, *sen*, *seo*, *seu*,	*lukE*	*t1398*
TC00516	M	TAN			*blaZ*, *dfrG*, *erm*(*C*)	*edinB*, *hlgA*, *hlgB*	*sak*, *scn*	*lukF-PV*, *lukS-PV*	*t355*
TC00522	M	TAN			*blaZ*, *dfrG*, *erm*(*C*)	*aur*, *edinB*, *hlgA*	*sak*, *scn*	*lukF-PV*, *lukS-PV*	*t355*
TC00523	M	TAN			*blaZ*, *dfrG*, *erm*(*C*)	*aur*, *edinB*, *hlgA*, *hlgB*	*sak*, *scn*	*lukF-PV*, *lukS-PV*	*t355*
BC00303	M	BAG	152	152	*dfrG*, *erm*(*C*), *tet*(*K*)	*aur*, *edinB*, *hlgA*, *hlgB*	*sak*, *scn*	*lukF-PV*, *lukS-PV*	*t355*
BC00306	M	BAG			*blaZ*, *dfrG*, *erm*(*C*), *tet*(*K*)	*edinB*, *hlgA*, *hlgB*	*sak*, *scn*	*lukF-PV*, *lukS-PV*	*t355*
MC00105	M	MOR			*blaZ*, *dfrG*, *tet*(*K*)	*edinB*, *hlgA*, *hlgB*	*sak*, *scn*	*lukF-PV*, *lukS-PV*	*t355*
TH00405	HWA	TAN			*blaZ*, *tet*(*K*)	*hlgA*, *hlgB*	*sak*, *scn*	*lukF-PV*, *lukS-PV*	*t11429*
HB00604	HWA	BAG			*blaZ*, *dfrG*, *erm*(*C*)	*aur*, *edinB*, *hlgA*, *hlgB*	*sak*, *scn*	*lukF-PV*, *lukS-PV*	*t355*
TC00402	M	TAN			*blaZ*, *dfrG*, *erm*(*C*)	*aur*, *edinC*, *hlgA*, *hlgB*, *hlgC*, *splA*, *splB*	*sak*, *scn*, *seg*, *sei*, *sem*, *sen*, *seo*, *seu*, *eta*, *etb*	*ND*	*t272*
DABST013	HNA	DSM	121	121	*blaZ*, *dfrG*, *erm*(*C*)	*aur*, *edinC*, *hlgA*, *hlgB*, *hlgC*, *splA*	*sak*, *scn*, *seg*, *sei*, *sem*, *sen*, *seu*, *eta*, *etb*	*ND*	*t272*
DADST045	HNA	DSM			*blaZ*, *dfrG*, *erm*(*C*), *fosB*, *tet*(*K*)	*aur*, *edinC*, *hlgA*, *hlgB*, *hlgC*, *splA*, *splB*	*sak*, *scn*, *sei*, *sem*, *sen*, *seo*, *seu*, *eta*, *etb*	*ND*	*t272*
DADST013B	HNA	DSM			*dfrG*, *erm*(*C*)	*aur*, *hlgA*, *hlgB*, *splA*, *splB*	*sak*, *scn*, *sei*, *eta*	*ND*	*t272*
BC00310	M	BAG			*blaZ*	*aur*, *edinB*, *hlgA*, *hlgB*, *hlgC*	*ND*	*lukE*	*ND*
BC00201	M	BAG	7845	5477-like	*blaZ*, *str*, *tet*(*K*)	*aur*, *edinB*, *hlgA*, *hlgB*, *hlgC*, *splA*, *splB*	*ND*	*lukE*	*t18853*
BC00410-2	M	BAG	7847		*qacG*	*aur*, *hlgA*, *hlgB*, *hlgC*, *splA*, *splB*, *splE*	*ND*	*lukE*	*ND*
TC00505	M	TAN	7848	5477-like	*tet*(*K*)	*aur*, *edinB*, *hlgA*, *hlgC*, *splA*, *splB*	*sei*, *sem*, *sen*, *seo*	*lukE*	*t528*
HK2002	HWA	BAG	22		*blaZ*	*aur*, *hlgA*, *hlgB*, *hlgC*	*sak*, *scn*, *seg*, *sei*, *sem*, *sen*, *seo*, *seu*, *tst*	*ND*	*t223*
BH00404	HWA	BAG	243		*ND*	*aur*, *hlgA*, *hlgB*, *hlgC*, *splE*	*seg*, *sei*, *sem*, *sen*, *seo*, *seu*	*lukF-PV*, *lukS-PV*	*t021*
MH00801	HWA	MOR	8		*aac*(*6*’)*-aph*(″), *blaZ*, *dfrG*, *erm*(*C*), *mecA*, *qacD*, *tet*(*K*)	*aur*, *hlgA*, *hlgB*, *hlgC*, *splA*, *splB*, *splE*	*sak*, *scn*, *seb*, *sej*, *sek*, *seq*, *ser*	*lukD*, *lukE*	*t1476*
DADST035	HNA	DSM	72		*blaZ*, *dfrG*	*aur*, *hlgA*, *hlgB*, *hlgC*, *splA*, *splB*, *splE*	*sak*, *scn*, *sec*, *seg*, *sei*, *sel*, *sem*, *sen*, *seo*, *seu*, *tst*	*lukD*, *lukE*	*t148*

Key: CC = clonal complex, ST = sequence type, AMR = antimicrobial resistance, *Spa* = Staphylococcal protein-a, ND = not determined, ID = identification, HWA = human with animal contact, HNA = human with no animal contact, BAG = Bagamoyo, MOR = Morogoro, TAN = Tanga.

## Data Availability

Data supporting reported results will be submitted to Ifakara Health Institute’s open access data repository under this article’s name. https://data.ihi.or.tz/index.php/catalog/Interventions (accessed on 14 March 2023).

## Supplementary Materials

The following supporting information can be downloaded at: https://www.mdpi.com/article/10.3390/microorganisms11061505/s1, Accession numbers assigned to the sequence after their submission to the European Nucleotide Archive has been provided.
